# NETosis and pyroptosis of immune cells in sepsis

**DOI:** 10.1515/jtim-2025-0035

**Published:** 2025-08-12

**Authors:** Ali Muhammad Kashif, Yang Ouyang, Yongqing Li, Baihong Pan

**Affiliations:** Department of Vascular Surgery, Xiangya Hospital, Central South University, Changsha, Hunan Province, China; Hunan Clinical Research Center for Vascular Intervention, Changsha, Hunan Province, China; Department of Surgery, University of Michigan Medical School, Ann Arbor, MI, USA

**Keywords:** NETosis, pyroptosis, sepsis

## Abstract

Sepsis, a potentially fatal illness marked by an abnormal host immune response to an infection, continues to be a major global health issue. This study explores the role of pyroptosis and neutrophil extracellular traps (NETs) and their complex interactions, two dominant immune cell death, in the setting of sepsis. The review begins by explaining sepsis and its strong dependence on the immune system. It then delves into the basic mechanisms of neutrophil extracellular trap associated death (NETosis) and pyroptosis, examining their functions in inflammation and host defense. Going further, it looks at the dysregulated immune response in sepsis, with a focus on neutrophils and other immune cells and their critical role. We analyze in detail two types of cell death: pyroptosis and NETosis, both of which are closely examined for their roles in the development of sepsis, particularly the escalation of pathophysiology. Additionally, the study looks into the complex interactions between pyroptosis and NETosis, illuminating possible mechanisms for control as well as their cooperative effects. The report concludes with a summary of the major findings and recommendations for future therapy approaches and research prospects. This review adds to our knowledge of the pathophysiology of sepsis by thoroughly analyzing the roles of NETosis and pyroptosis in sepsis and providing possible directions for therapeutic intervention.

## Graphical Abstract

**Figure:**
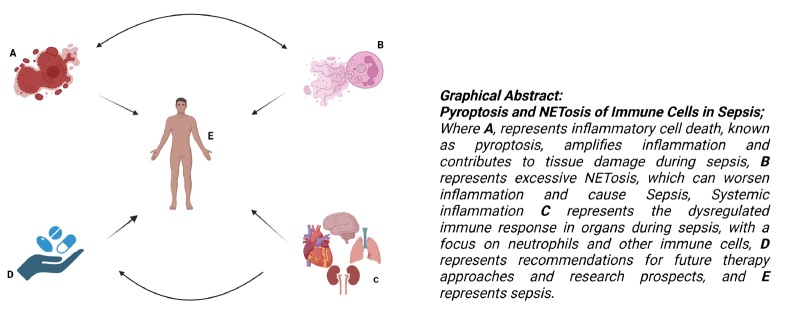

## Introduction

Sepsis is a severe, occasionally widespread inflammation and fatal organ failure that results from an unbalanced host response to various infections.^[1]^ The mortality rate for septic patients is still 25%–30% due to the rapid progression of sepsis, and it can reach 40%–50% in cases of septic shock.^[2]^ Thus sepsis is a significant worry in the critical care field today.^[3]^ Immune cells play a crucial role in the pathogenesis of sepsis, and the two forms of cell death, known as neutrophil extracellular trap associated death (NETosis) and pyroptosis, have been implicated in the progression of sepsis.^[4]^ NETosis is a distinct type of cell death that is mostly linked to neutrophils, a subset of white blood cells. These threads create extracellular traps that can catch and kill microorganisms, but they can also damage the body when inflammation gets out of hand.^[5]^ Dysregulated neutrophil cell death may be dangerous in septic patients because it increases the risk of immune-related organ failure, which lowers body defenses and makes patients more susceptible to hospital-acquired infections. Neutrophils have therefore changed from being strong antibacterial agents to possibly dangerous mediators of organ failure and tissue damage. Additionally, Immune cells including dendritic and macrophage cells, may also undergo pyroptosis which is a highly inflammatory kind of programmed cell death, in reaction to particular microbial infections.^[6]^ During pyroptosis, a group of proteins called inflammasomes are activated. These proteins serve as intracellular sensors for pathogen-associated molecular patterns (PAMPs) or danger-associated molecular patterns (DAMPs).^[7]^ However, the pathophysiology of sepsis is still not entirely understood, and the absence of effective treatment options contributes to the shockingly high fatality rate. Many advancements and improvements have been made in the past ten years as research on this condition has transitioned from systemic inflammatory response syndrome to multiple organ dysfunction syndrome.^[8]^

## The pathophysiology of sepsis

The most common cause of death in intensive care units is pathogen infection, which can lead to sepsis, a potentially fatal condition. However, new research suggests that immunological dysfunction and sepsis are largely driven by massive immune cell deaths.^[9]^ The primary cause of sepsis is an improper balance between the host’s innate and adaptive immune responses. This procedure involves a multitude of immune cells, such as lymphocytes, mononuclear macrophages, and neutrophils. Immune cells identify pathogens, consume them, and then release cytokines to either directly or indirectly recruit or activate other cells. Several unique kinds of cell death, distinct from apoptosis, such as autophagy, immune cell-extracellular trap formation, and pyroptosis, are crucial in the advancement of sepsis. Through the three types of cell death listed above, immune cells might start “self-sacrifice” in order to either kill or defend against infections. Nevertheless, the precise functions and workings of immune cell self-sacrifice in sepsis remain unclear.^[10]^

## Immune response to infection

Understanding the typical acute inflammatory response is vital to comprehending the dysregulated immune response that underpins the pathophysiology of sepsis. A number of plasma enzyme cascades are triggered, a variety of inflammatory cells are generated, and chemical mediators from cells and plasma are released when an infection or injury occurs. The leukocytes’ emigration through the porous capillary walls and subsequent entry into the injured area trigger the immune response at the same moment. Neutrophils locate and break down bacteria and cellular waste using a process known as chemotaxis, which is guided by substances in the tissues. After migrating to the injured location, monocytes grow larger and transform into macrophages, which then engulf bacteria and phagocytize foreign objects. Histamine is released when mast cells within the tissues degranulate, which also triggers the production of other inflammatory mediators. Specifically, histamine increases capillary permeability and induces further vasodilation.^[11]^

The immune system’s original purpose is to protect the host against the enormous array of dangerous microorganisms that are constantly changing. Additionally, immunity helps the recipient eliminate allergens and hazardous chemicals that may have entered through mucosal surfaces accidentally. The most prevalent white blood cell in the bloodstream, neutrophils act as the host’s first line of defense against pathogen invasion. These are short-lived, terminally differentiated cells that come out of the bone marrow prepared to respond to infections.^[12,13]^ The neutrophil has a variety of defense mechanisms it can use to ensure the best possible elimination of a hazard once it has detected a foreign molecule or endogenous threat. These include the capacities for degranulation, phagocytosis, and the generation of reactive oxygen species (ROS). In order to maximize the host’s immunological response and warn other nearby cells of the threat, neutrophils can also release chemokines and cytokines.^[14]^ Neutrophils also employ decondensed chromatin adorned with antimicrobial peptides as a means of defense. This process is known as neutrophil extracellular trap (NET) formation, and it allows the neutrophil to ensnare the pathogen.^[15]^

## Dysregulated immune response in sepsis

The typical inflammatory response changes with sepsis. Infection sets off a typical inflammatory response, which thereafter causes the release of certain cytokines. These cytokines trigger other chemical inflammatory mediators to call for additional assistance. The inflammatory mediators initiate a sequence of events known as cascades that give rise to and maintain the inflammatory reply. Sepsis suggests a potentially fatal organ failure brought on by an infection-related dysregulated host response. A clinical definition of organ dysfunction in sepsis is a two-point increase in the Sequential Organ Failure Assessment (SOFA) score or above. Patients with suspected infections in a hospital may have an approximate 10% mortality risk if their SOFA score is ≥2. Septic shock, which is characterized as severe metabolic, cellular, and circulatory dysregulation from underlying sepsis, can result from sepsis. The pathological process of sepsis is extremely complex, as previous research has shown, and this continues to be a major concern in critical care medicine today.^[3,16]^ Furthermore, the pathophysiology and progression of sepsis are significantly influenced by the immune response. In the early stages of sepsis, immune cells release cytokines and inflammatory mediators to try and eliminate possible pathogens; in the later stages, immune-suppressive mechanisms emerge, leading to immunological dysfunction.^[17]^

## Role of neutrophils and other immune cells in sepsis

Volker Brinkmann stimulated neutrophils with lipopolysaccharide (LPS), phosphor myristate (PMA), and IL-8 for the first time in 2004. They discovered that active neutrophils underwent a process known as “NETosis”, in which they flattened down and created noticeable extracellular structures known as “NETs”. In addition to effectively eliminating bacteria, the fibrous structure of NETs can operate as a physical barrier to stop the spread of bacteria. On the other hand, they noticed that the immune system could be harmed by an excessive amount of extracellular histone complex exposure, suggesting that NETosis was a two-edged sword for the immune system.^[18]^

Pyroptosis is a lytic type of planned cell death carried out by gasdermins, which are proteins that generate pores in cells. Gasdermin A (GSDMA), GSDMB, GSDMC, GSDMD, GSDME, and GSDMF (PJVK/DFNB59) are members of the gasdermin family.^[19]^ Gasdermins have a linker connecting the C-terminal domain to the N-terminal domain, which is lipophilic and naturally makes pores. In a steady state, the C-terminal domain suppresses the N-terminal domain’s ability to produce pores. Specific proteases, activated in response to different signals, relieve intramolecular inhibition by cleaving the linker region that separates the N- and C-terminal domains. The N-terminal domain then translocates to the plasma membrane and attaches itself to the acidic phospholipids in the cytoplasmic leaflet, including phosphoinositides. Following this, the N-terminal domain oligomerizes and creates pores on the plasma membrane that resemble rings. The buildup of gasdermin pores ultimately causes cell lysis.

## Neutrophil extracellular traps

### Definition and formation of NETs

**Figure 1:**
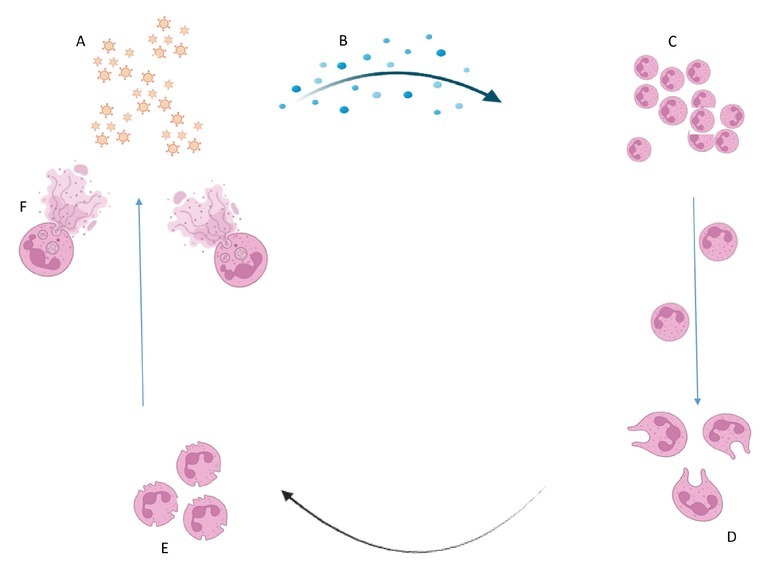
Formation of NETs. Where A, represents infection site; B, represents Chemotactic signals guiding neutrophils to the infection site; C, represents Neutrophil Activation and Granule Degranulation; D, represents neutrophils membranes protrusions or pseudopodia formation; E, represents degranulation of neutrophils, and F represents neutrophil undergoing NETosis, with the chromatin (DNA) and antimicrobial proteins (like NE, proteinase 3) being expelled from the neutrophil.

As the initial line of defense against invasive infections, neutrophils are essential to innate immunity. They are widely distributed, and when they receive chemotactic signals, they transmigrate to the infection sites. When activated neutrophils get to the site of the infection, they fight pathogens in a number of ways, including phagocytosis, oxidative burst, and “NETosis”, the process of making and releasing neutrophil extracellular traps (Figure 1). NETs are web-like structures made up of anti-microbial proteins such as serine proteases cathepsin G, proteinase 3, and neutrophil elastase (NE) and the neutrophil’s own DNA. When depolymerized chromatin and intracellular granular proteins are released, neutrophils activate, capture, and eliminate pathogens. This process, known as NETosis, would cause neutrophils to perish.^[10]^

One of the dominant mechanistic signalling pathways of NET formation is histone citrullination. When arginine and methylarginine in histones are changed to citrulline, the neutrophil chromatindecondensates, which leads to the growth of NETs. NETs are then expelled through NETosis. Additional information regarding the induction of NETosis was provided by studies linking autophagy to NET formation in PMA-induced neutrophils, both autophagy and production of ROS were required for NET formation. Interestingly, ROS production and autophagy occurred independently of each other; however, activation of both pathways was required for efficient chromatin decondensation. Additionally, pharmacological inhibition of the mTOR pathway enhanced autophagy and accelerated NET formation in neutrophils.^[20]^ As a result of more research, it has been discovered that additional immune cell types are capable of causing extracellular traps to form, which release histones and DNA into the host tissue in a directed cell death process. NETs play a significant role in the regulation of infections and are a component of the innate immune response. Although NETs are mainly thought of as a defense mechanism against a variety of pathogens, there is mounting evidence linking NETs to the pathophysiology of organ dysfunction.^[21]^

### Functions of NETs in host defense

As the initial line of defense in the innate immune system against microorganisms, neutrophils can eradicate invasive pathogens in many ways. The most common defensive mechanism, phagocytosis, is a well-known process that includes engulfing the prey inside the phagosome, an internal compartment. The phagosome then develops by fusing with other preexisting neutrophil granules, forming an unfriendly chamber that is loaded with harmful amounts of proteases, antibiotic peptides, and other microbicidal substances, most notably ROS. The NOX2-containing NADPH-oxidase, an electron-transporting enzyme system, produces these ROS by converting NADPH to superoxide. The entrapped prey is often killed by the combined effects of the ROS and the protein/peptide antimicrobials.^[22]^

Another defense mechanism used by neutrophils to capture and potentially eliminate microorganisms is the creation of what are known as NETs.^[15]^ The nuclear DNA of the neutrophil is what makes up the extracellular cobweb-like fibrils known as NETs. Myeloperoxidase (MPO), proteases, histones, and antimicrobial peptides are all covered in these fibrils, which are then actively expelled from the cells through a process known as NETosis. This remarkable method of cell death has been linked to viral diseases, but it has also been demonstrated to have significance in non-infectious situations, such as autoimmune disorders, where the immune system is exposed to nuclear and granule material.^[15,23]^

## Netosis in sepsis

### Introduction to NETs

Brinkmann *et al*. originally described NETs as a host defensive mechanism against bacteria.^[15]^ NETs can catch and kill germs by using proteinases and MPO, which stops them from getting into the bloodstream and nearby tissues.^[15,24,25]^ However, uncontrolled NET formation on wounded vascular endothelial cells in sepsis promotes platelet aggregation and coagulation at the sites of attachment.^[26, 27, 28]^

### NETs in Physiological settings

#### Interaction Between Platelets and Neutrophils in Sepsis

A 2007 study by Clark *et al*. demonstrated how platelets and neutrophils interact during sepsis, leading to the creation of NETs and improved bacterial entrapment in blood vessels.^[29]^ Neutrophils exhibit a longer life span and decreased migration during sepsis. This restricts them to blood vessels, whereupon they release cytokines, ROS, and NETs, which cause severe vascular inflammation.^[30,31]^ Specifically, pro-inflammatory and pro-angiogenic responses in endothelial cells are triggered by neutrophils and NETs, further deregulating the immune system.^[32,33]^ An elevated rate of glycolysis is seen in activated endothelial cells, which intensifies oxidative stress and inflammation.^[34,35]^

#### Effects of NETs on Endothelial Cells and Vascular Inflammation

NETs,^[15,36]^ one type of neutrophil activation may promote intravascular coagulation, damage vascular endothelial cells, and prevent bacterial movement in the bloodstream, all of which can result in the formation of immunothrombi.^[37, 38, 39]^ Neutrophil-endothelial contact increases in sepsis to encourage neutrophil infiltration into tissues.^[40]^ Increased NET formation is caused by the interaction between neutrophils and endothelial cells; this increased NET formation is partially reliant on IL-8 produced from activated endothelial cells.^[41]^ Long-term neutrophil co-cultivation with endothelial cells caused endothelial cell damage; this damage is attributed to NETs because co-incubation with DNase or NAPDH oxidase inhibitors reduced the damage.^[41]^ Human, murine, and canine neutrophils respond to LPS in ex vivo environments by going through a limited degree of NETosis.^[42, 43, 44]^ However, both human and murine neutrophils release significant numbers of NETs when LPS-activated platelets are present, suggesting that platelet-neutrophil interaction plays a major role in sepsis-associated NETosis. It was initially discovered by Clark *et al*. that platelet Toll-like receptor 4 is necessary for platelet-mediated NETosis in humans that is caused by LPS.^[29]^

## Pyroptosis

### Definition and formation of pyroptosis

**Figure 2:**
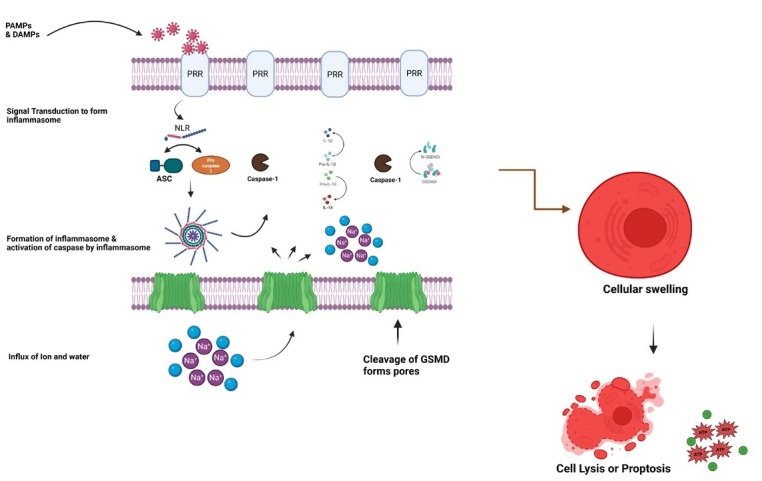
Formation of cell pyroptosis.

Immune cells use pattern recognition receptors to actively search for PAMPs, pathogen-induced cellular changes, and DAMPs in both the extracellular and intracellular compartments.^[45]^ These receptors can trigger an inflammatory reaction by increasing the production of proinflammatory cytokines and chemokines (Figure 2). They can also initiate homeostatic responses, such as autophagy, which helps remove PAMPs and destroy damaged organelles. In addition, inflammasomes, which are specialized sensors of the innate immune system, trigger pyroptosis. Inflammasomes are complex assemblies composed of various components, including sensor molecules such as the nod-like receptor (NLR) family pyrin domain containing 3 (NLRP3), Absent in Melanoma 2 (AIM2), NLR family CARD domain containing 4 (NLRC4), and others. These sensor molecules are accompanied by an adaptor protein called apoptosisassociated speck-like protein containing a CARD (ASC), as well as an effector protease known as caspase-1. These oligomeric complex acts as a platform to cleave pro-Caspase-1 zymogen into active form. The presence of microbial components or cellular disturbances caused by infection triggers the construction of inflammasomes, resulting in the self-cleavage of caspase-1 into its active form.^[46, 47, 48]^ Activated caspase-1 leads to DNA fragmentation and to cell lysis through different signal pathways. Caspases can activate nuclease and subsequent DNA fragmentation independent of degradation of inhibitor caspase-activated deoxyribonuclease (ICAD). Caspase-1 also facilitates the process of converting immature forms of interleukin-1β (IL-1β) and interleukin-18 (IL-18) into their mature counterparts, which are released easily through cell lysis and promote inflammation. Significantly, GSDMD has been identified as the substrate of caspase-1.^[49,50]^ GSDMD can also be targeted by other caspases, namely caspase-11 in rats and caspase-4/5 in humans. These caspases work as detectors for gram-negative bacterial LPS found in the cytosol.^[51, 52, 53, 54]^ The cleavage of GSDMD by Caspase-1/11/4/5 releases the N-terminal domain, leading to the perforation of the plasma membrane.^[55,56]^ The development of pores by GSDMD results in the entry of ions, swelling due to osmotic pressure, and ultimately the lysis of cells, or pyroptosis, letting go of cellular contents such as proinflammatory cytokines (IL-1β and IL-18), ATP and other alarmins.

### Pyroptosis in inflammation and immune response

Pyroptosis is an intensely inflammatory kind of programmed cell death that has a critical function in triggering inflammation and immunological responses. They are involved in regulating and activating caspase-1 or caspase-4/5/11, resulting in the formation of holes in the plasma membrane. This causes the cell to break down and release pro-inflammatory chemicals.^[54]^ These are primarily associated with the innate immune system, serving as a safeguard against microbial infections.

In the end, the cell goes through pyroptosis, which let go of its internal contents. These include pro-inflammatory cytokines like IL-1β and IL-18, as well as warning signals like high-mobility group box 1.^[57]^ These chemicals attract and stimulate immune cells, intensifying the inflammatory response and facilitating the elimination of infections. The secretion of IL-1β and IL-18 plays a crucial role in beginning and maintaining inflammation. These cytokines stimulate nearby cells, such as endothelial cells and immune cells, to produce a variety of other pro-inflammatory cytokines and chemokines. ^[52]^ This enhances the inflammatory response and attracts immune cells to the location of infection or tissue damage. IL-1β additionally aids in the stimulation of adaptive immunity by facilitating the differentiation of T cells and the generation of antibodies. Pyroptosis can also directly aid in host defense by removing intracellular infections. The process of pyroptosis, which is initiated by caspase-4/5/11, is activated upon the recognition of cytosolic LPS originating from Gram-negative bacteria.^[58]^ This type of pyroptosis enables the liberation of bacteria that are inside cells into the area outside the cells, which helps phagocytes identify and eliminate them.

Pyroptosis mainly happens in macrophage and dentric cells. However, research have identified other cell types going through pyroptosis, such as neutrophils through activating a non-canonical inflammasome pathway through P2X7 receptors. Neutrophils are highly microbicide with short life span. Through pyroptosis, chemo attractive capacity and phagocytosis, few pathogens can survive and replicate.

Although pyroptosis serves as a potent defense mechanism, its dysregulation can contribute to the development of different illnesses. Excessive or prolonged pyroptosis can result in tissue damage and the development of chronic inflammation.^[59]^ Moreover, autoinflammatory diseases and immunodeficiencies have been associated with alterations in the genes that code for inflammasome components or pyroptosis-related proteins.

## Crosstalk between netosis and pyroptosis

The term “crosstalk” describes the possible regulatory relationships and interplay between NETosis and pyroptosis, two types of immune responses and cell death. During NETosis, the recruited neutrophils release cytotoxic substances and enzymes in response to pathogens, phagocytose pathogens, and release NETs to carry out host defensive functions.^[60]^ The activation of neutrophils triggers NETosis, which also coincides with changes in intracellular calcium concentration, an increase in ROS, and the activation of kinase signalling cascades that aid in the synthesis of NETs.^[61]^ It has been documented that the same infections that cause NETosis can cause pyroptosis in different kinds of periodontal tissue cells.

Additionally, Inflammasomes are also a major part of the innate immune system and consist of sensors, adaptors, and effectors which serve as a platform within cells to identify various pathogens and harmful signals. Most of the inflammasomes that have been found so far are NLRP1, NLRP3, NLRP6, AIM2, Pyrin, and CARD8.^[62]^ Upon activation, the effectors then cut downstream effectors like pro-IL-18, pro-IL-1β, and GSDM family members. These are then released from cells to start pyroptosis and the inflammatory cascade.^[47]^

However, while inflammasomes are mostly expressed in myeloid cells, such as macrophages and monocytes, there is mounting evidence that they are also essential components of the adaptive immune system, particularly in lymphocytes. Inflammasomes are expressed and activated in lymphocytes, and lymphocytes in turn regulate inflammasome activity in the adaptive immune system. This relationship between inflammasomes and lymphocytes acts as a bridge for communication between the innate and adaptive immune response, resulting in fine control of immune protection. This study examines the crosstalk that inflammasomes facilitate between the innate and adaptive immune systems, with a particular emphasis on the relationship between inflammasomes and lymphocytes.

## Synergistic effects of netosis and pyroptosis in sepsis

The synergistic effects of NETosis and pyroptosis play a significant role in the pathogenesis of sepsis. NETosis creates NETs, composed of chromatin and antimicrobial proteins. Because of their ability to capture and eliminate pathogens, these NETs are helpful during the early stages of an infection. Nevertheless, there may be negative consequences from increased NET creation and decreased NET clearance. In sepsis, NETs components stimulate immune cells and cause the production of inflammatory mediators such as chemokines and cytokines, which in turn promote systemic inflammation.^[63]^ Also, reserch suggests that NETs can exerbate pyroptosis. For example, NETs originated DNA is able to augment pyroptosis *via* AIM2 inflammasome activation, and NETs originated NE can cleave and activate GSDMD which is a key executive protein of pyroptosis.^[64,65]^

On the other hand, pyroptosis is a type of programmed cell death that is highly inflammatory. Inflammasomes, multiprotein complexes that trigger the cleavage and secretion of pro-inflammatory cytokines including IL-18 and IL-1β, are what define them. In sepsis, these cytokines help to magnify the inflammatory response.^[66]^ These cytokines can then trigger NETosis. Furthermore, researchers have suggested that pyroptosis is an upstream regulator of NETosis, and GSDMD, the key executive protein of pyroptosis, facilitates NETosis.^[65,67]^ More pro-inflammatory cytokines are released, and pyroptosis is enhanced when NETs trigger inflammasomes., which can lead to an inflammatory cycle that amplifies itself again.^[68]^ Moreover, NETs have the direct ability to cause immune cells to undergo pyroptosis, which intensifies the inflammatory response.^[69]^ The interplay between NETosis and pyroptosis exacerbates sepsis-related immunological dysregulation.

Therefore, understanding the synergistic effects of NETosis and pyroptosis provides new insights into sepsis pathogenesis and potential therapeutic targets. Targeted interventions aimed at regulating these processes could help mitigate immune dysregulation and improve patient outcomes. However, further research is needed to fully elucidate the underlying mechanisms and identify specific therapeutic strategies.^[70]^

## Modulation of netosis and pyroptosis pathways

Modulation of NETosis and pyroptosis pathways is an area of active research in the field of immunology. Immunological research is currently being conducted in the domains of NETosis and pyroptosis pathway modification. Both pyroptosis and NETosis are specialized types of cell death that are crucial for inflammation and immunological responses. Excessive NET development can lead to tissue damage and autoimmune disorders.^[15,63]^ Additionally, a variety of stimuli can cause pyroptosis, an inflammatory type of programmed cell death. Cell lysis and the release of pro-inflammatory cytokines (such as IL-1β and IL-18) are its defining features. Pyroptosis is modified by controlling downstream signalling pathways and inflammasome activation.^[48,71]^

## Therapeutic strategies targeting netosis and pyroptosis

Circulating NETs are observed in individuals with sepsis, which is often accompanied by severe multi-organ failure and unfavourable clinical outcomes.^[72,73]^ In contrast, an unmanageable rise in NET formation, along with the failure to remove and break down debris and contents resulting from cell death, including cell-free DNA, results in heightened expression of inflammatory mediators such as tumor necrosis factor α in these individuals. There have been reports indicating that citrulline histones produced from NETs can function as DAMPs. They achieve this by stimulating the production of inflammatory cytokines, impairing the function of endothelial cells through cytotoxicity, and increasing the formation of ROS. As a consequence, this leads to damage to multiple organs.^[74,75]^ However, pyroptosis, which is a type of programmed cell death that is extremely inflammatory and is activated by inflammasomes, has a crucial function in protecting the host against intracellular pathogens. However, if it is not properly managed, it can worsen tissue damage and inflammation.^[76]^ The dysregulation of NETosis and pyroptosis has been linked to the development of various diseases, such as autoimmune disorders (*e.g*., lupus, rheumatoid arthritis), infectious diseases (*e.g*., sepsis), and inflammatory conditions (*e.g*., atherosclerosis, cancer).^[77,78]^ As a result, there has been significant interest in using therapeutic strategies that focus on these pathways as prospective interventions to reduce disease severity and improve patient outcomes. NETs have been linked to the development of many inflammatory and infectious conditions. Strategies aimed at inhibiting NETosis have the potential to reduce tissue damage and inflammation associated with these disorders. Kenny *et al*. showed that various stimuli activate distinct pathways that result in the creation of NETs, indicating possible targets for inhibiting NETosis.^[77]^ In addition, a study also discovered that inflammatory caspases can operate as innate immunological receptors for intracellular LPS. This finding implies that it may be possible to regulate NETosis by targeting caspase activation.^[76]^ Overall, these results emphasize the need for establishing therapeutic ways to inhibit NETosis and decrease the inflammatory response in various illness scenarios. Pyroptosis is a type of programmed cell death that is highly inflammatory. It is important for the body’s defense against intracellular infections but can cause more tissue damage and inflammation if not well regulated. Shi *et al*. found that inflammatory caspases act as innate immunological receptors for LPS inside cells, highlighting their important role in starting pyroptosis.^[76]^ Suppressing the activity of inflammatory caspases is a promising approach to controlling pyroptosis and reducing its harmful consequences. By focusing on certain caspases’ activation or activity, it might be possible to lower the inflammatory response connected to pyroptosis while still letting it do its important job of protecting the host.

## Current therapeutic approaches for sepsis

Peptidylarginine deiminases (PADs) show promising therapeutic benefits for acute lung injury produced by sepsis.^[79]^ In a sepsis model, PAD-knockout mice enhanced survival and reduced the degree of organ failure and progression of sepsis.^[80]^ NE, also known as NE, is a protease that is stored in neutrophils and is released into the extracellular environment during inflammatory responses or the creation of NETs.^[81]^ NE plays a role in the creation of NETs, and efficiently blocking NE minimizes damage to lung tissue.^[82]^ Sivelestat is a small-molecule inhibitor of NE that can inhibit NET formation *in vitro*, ease clinical symptoms of lung injury, lower inflammatory cytokines in the blood, and strengthen the body’s defense mechanism.^[83]^ Sivelestat improves acute lung damage in patients with sepsis.^[84]^ DNase can alter the structure of NET and lower NET levels.^[85]^ Dwivedi *et al*. assessed 80 patients with sepsis and found that cell-free DNA levels were a better predictor of critical care unit death compared to standard scoring systems.^[86]^ A promising way to treat sepsis involves combination therapy targeting both NETosis and pyroptosis. It may be possible to create synergistic effects and improve patient outcomes by simultaneously regulating various inflammatory pathways. However, the development of such therapeutics requires a full understanding of the interplay between NETosis and pyroptosis, as well as the identification of critical molecular targets for intervention. Several studies have established the efficacy of combination treatments in preclinical models of sepsis. For example, reduction of NETosis by DNase I and blocking of pyroptosis using caspase inhibitors have been demonstrated to minimize organ damage and enhance life in septic animals.^[54,87]^

## Conclusion

In conclusion, sepsis remains a significant global medical challenge, causing high rates of illness and death. Our analysis emphasizes the crucial role of immune response dysregulation in sepsis. We have identified the interaction between two processes, NETosis and pyroptosis, which play a key role in the development of sepsis. NETosis involves the release of chromatin and antimicrobial proteins from neutrophils, while pyroptosis is an inflammatory form of programmed cell death. These processes contribute to tissue damage and systemic inflammation in sepsis. Understanding this interaction opens new avenues for targeted therapies, although further research is needed. Collaboration and translational research are essential to improving patient outcomes in sepsis.
